# Effect of citric-acid dialysate on the QTC-interval

**DOI:** 10.1038/s41598-021-89083-w

**Published:** 2021-05-10

**Authors:** Karlien J. ter Meulen, Ben J. M. Hermans, Frank M. van der Sande, Bernard Canaud, Constantijn J. A. M. Konings, Jeroen P. Kooman, Tammo Delhaas

**Affiliations:** 1 Division of Nephrology, Department of Internal Medicine, Maastricht University Medical Center, PO Box 5800, 6202AZ Maastricht, The Netherlands; 2 Division of Nephrology, Department of Internal Medicine, Catharina Hospital, Eindhoven, The Netherlands; 3 Department of Biomedical Engineering, Maastricht University, Maastricht, The Netherlands; 4 Department of Cardiology, Maastricht University Medical Center, Maastricht, The Netherlands; 5 Cardiovascular Research Institute Maastricht (CARIM), Maastricht University, Maastricht, The Netherlands; 6 Fresenius Medical Care, Bad Homburg, Germany

**Keywords:** Haemodialysis, Nephrology

## Abstract

Lower dialysate calcium (dCa) concentration and dialysate citric-acidification may positively affect calcification propensity in serum of haemodialysis (HD) patients. However, the accompanying lower ionized blood calcium concentration may lead to a prolonged cardiac action potential, which is possibly pro-arrhythmic. The aim of this study is to investigate the influence of citric-acid dialysate on the QT-interval corrected for heart rate (QTc) compared to conventional dialysate with different dCa concentrations. We conducted a four-week multicentre, randomized cross-over trial. In week one and three patients received acetic-acid dialysate with a dCa of 1.50 mmol/l (A1.5), in week two and four acetic-acid dialysate with a dCa of 1.25 mmol/l (A1.25) or citric-acid dialysate (1.0 mmol/l) with a dCa of 1.50 mmol/l (C1.5) depending on randomization. Patients had continuous ECG monitoring during one session in week one, two and four. The data of 13 patients were available for analysis. Results showed a significant though limited increase of QTc with C1.5 (from 427 to 444 ms (start to end); p = 0.007) and with A1.25 (from 431 to 449 ms; p < 0.001), but not with A1.5 (from 439 to 443 ms; p = 0.13). In conclusion, we found that the use of C1.5 or A1.25 is associated with a significant prolongation of QTc which was however relatively limited.

## Introduction

Although lower dialysate calcium concentrations (dCa) may positively affect the calcification tendency in serum of haemodialysis (HD) patients^1^, it may also potentially have a prolonging effect on cardiac action potential^2–4^. In addition, the PACE study suggested that predialysis low serum potassium (K) and ionized calcium (iCa) are associated with higher risk prolongation of heart rate-corrected QT-intervals (QTc)^5^. HD is associated with a risk of prolongation of QTc^6,7^, which can be associated with increased mortality and sudden cardiac arrest^8,9^**.** QTc can be considered prolonged when ≥ 450 ms for men, and ≥ 460 ms for women^10^. International practice patterns are conflicting upon proposed dCa. Whereas a dCa of 1.25 mmol/l is more common in the United States, in Europe and Japan a dCa of 1.50 mmol/l is frequently used.

The most common dialysis fluid in bicarbonate (Bic) dialysis is based on a combination with acetate which even in small concentrations is hypothesized to have an effect on haemodynamic stability during dialysis^11–14^. Also used in Bic dialysis is the citric-acid dialysate (dCit) that showed a positive influence on the dialysis efficiency while haemodynamic stability was possibly improved^15,16^. Citrate chelates iCa and magnesium (Mg). Thus lower iCa effects cardiac repolarisation and eventually can lead to an increased QTc and higher risk on arrhythmia^2^.

A continuous QTc monitoring during acetate-free biofiltration with hemodiafiltration (HDF) session with different dCa (1.25 mM/l, 2 mM/L and profiled Ca) showed increased QTc for the lower dCa^17^. Floccari et al. performed a hourly ECG during three acetate-free HDF sessions with dCa of 1.75 mmol/l which showed a negative correlation of QTc with serum Ca levels at the end^4^. However, most studies comparing different dCa concentrations only addressed differences in QTc between the start and end of dialysis, whereas to the best of our knowledge the effect of dCa on QTc nor the effect of dCit on the QTc during conventional HD has not been studied yet. We studied the influence of conventional Bic dialysis combined with acetate-acid with dCa 1.50 mmol/l (A1.5), dCa 1.25 mmol/l (A1.25) and dCit with dCa 1.50 mmol/l (C1.5) on QTc during the complete dialysis sessions. The hypothesis was that QTc would increase during HD with C1.5 as compared to A1.5.

## Methods

### Study design

We have conducted a multicenter, randomized cross-over trial in two Dutch hospitals with the primary focus on the effect of the different dialysate concentrations on calcification propensity and calcium mass balance. These have been published elsewhere^1^. The study design has been published before^1^. Patients with an a priori QTc prolongation of ≥ 470 ms were excluded from participation. We did a sub analysis on this study to explore the influence of the dialysates used on QTc during the complete dialysis sessions. Researchers obtained written informed consent from the patients. The study was primarily approved by the Medical Research Ethics Committee (METC) of the Maastricht University Medical Center/Academic Hospital Maastricht (METC.151085) and secondary by the METC of Catharina hospital in Eindhoven. Both boards of directors gave approval. This study was prospectively registered in Dutch Trial Registry (NTR 5226) on the 23/04/2015. The study was monitored by Clinical Trial Center Maastricht and was conducted according to the principles of the declaration of Helsinki.

### Dialysate composition

Haemodialysis sessions were bicarbonate-based. All three dialysis fluids consisted of 138.0 mmol/l sodium, 0.5 mmol/l magnesium and 1.0 g/l glucose. The calcium concentration was either 1.25 mmol/l (A1.25) or 1.50 mmol/l (A1.5 and C1.5). Whereas A1.25 and A1.5 contained 3.0 mmol/l acetate, the acetate was replaced with 1.0 mmol/l citrate in C1.5. Levels of K (2–3 mmol/l) and Bic (provided with *Bibag*, range 30-36 mmol/l) were individualized, but the concentrations did not change during the study. All used dialysates are registered products that are common in daily practice. All patients had A1.5 as their regular dialysate.

### Electrocardiography analysis

The *Task Force Monitor* (TFM, CN Systems, Austria) was used to record ECG during dialysis sessions. To improve ECG signal-to-noise ratio, a median complex was constructed for every 2 min by aligning all complexes within these two minutes on the R-peak. QT-intervals were determined offline using an automated tangent approach by a custom-made algorithm in *MATLAB* (2017a, Mathworks, Natick, MA, USA)^18^. All ECG landmarks were checked manually by one observer who was blinded for the treatment. QTcs were calculated using Bazett’s formula^19^. The first median complex (i.e. first two minutes after start dialysis) was used as the baseline (QTc_Baseline_). For each hour of dialysis, median QTc (QTc_1h_, QTc_2h_ etc.) were calculated. All recordings were made during the second or third session of week one, two and four in order to omit the effects of a long interdialytic period on the haemodynamic response during HD.

### Statistical analysis

The derived data were analysed using IBM SPSS Statistics for Windows version 23.0 (IBM Corp. Armonk, NY, USA). Data was expressed as median with interquartile range [25th; 75th percentile]. Due to the small sample size, non-parametric testing was applied. Friedman was applied to investigate differences within and between the dialysates; in case of statistical significance Wilcoxon Signed Rank was used to investigate the change. Correlations were tested by Spearman’s rho. A p-value < 0.05 was considered statistically significant. No additional correction for multiplicity was used.

## Results

Out of the 20 patients, 7 patients were excluded because of missing recordings (n = 3) or cardiologic disorders that may affect QTc (atrial fibrillation (n = 1), repolarisation disorders (n = 2) and frequent ventricular extrasystoles (n = 1)). Patients had a median age of 69 [51; 75] years, 7 were male (53.8%) and the median dialysis vintage was 25 [7.5; 66] months. Causes of renal failure in the remaining 13 patients were renal vascular disease (n = 5; 38.5% of which 2 were due to hypertension), diabetes mellitus type 2 (n = 2; 15.4%), congenital renal dysplasia with urinary tract malformation (n = 1; 7.7%), membrano-proliferative glomerulonephritis (n = 1; 7.7%), chronic renal failure aetiology unknown (n = 1; 7.7%) and other (n = 3; 23.1%). A total of 12 (92.3%) patients were diagnosed for hypertension and 3 (23.1%) were current smokers.

### QTc

**Figure 1:**
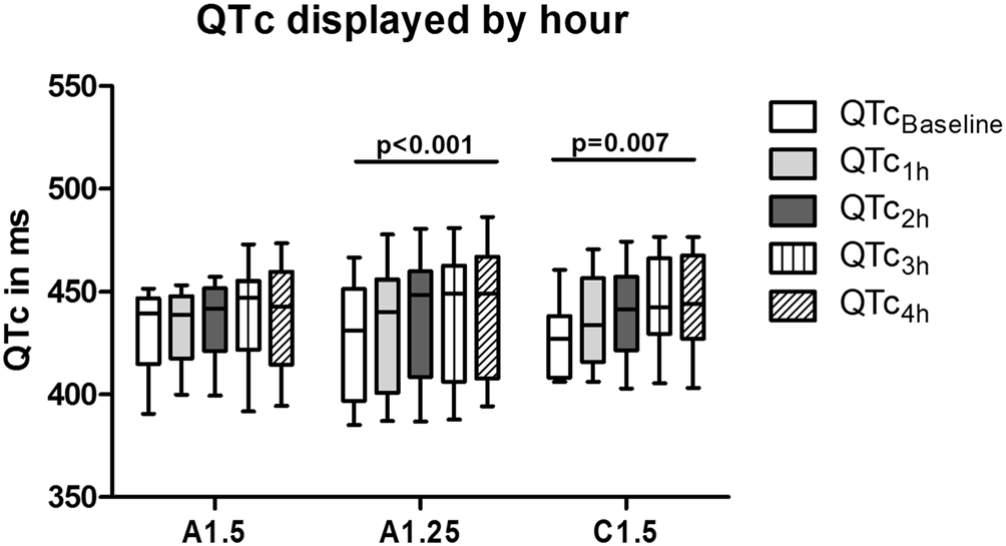
QTc displayed per hour**:** Data are expressed as median with minimum and maximum. A1.5 = acetic-acid dialysate with calcium concentration 1.50 mmol/l. A1.25 = acetic-acid dialysate with calcium concentration 1.50 mmol/l. C1.5 = citric-acid dialysate with calcium concentration 1.50 mmol/l. QTc_Baseline_ = QTc of the first median complex (first 2 min of haemodialysis). QTc_1h_, QTc_2h_, QTc_3h_, QTc_4h =_ median QTc of respectively the first, second, third and fourth hour of haemodialysis. *p-value was measured with Friedman’s test.

Data are summarized in Table 1 and Fig. 1.

**Table 1:** Overview of QTc and RR per dialysate.

		A1.5	A1.25	C1.5	p-value
QTc in ms (N = 13)	QTc_Baseline_	439 [415; 447]	431 [397; 451]	427 [408; 438]	0.37
	QTc_1h_	439 [418; 448]	440 [401; 456]	434 [416; 456]	0.23
	QTc_2h_	442 [421; 452]	448 [409; 460]	441 [422; 457]	0.50
	QTc_3h_	447 [422; 455]	449 [406; 463]	443 [430; 466]	0.29
	QTc_4h_	443 [415; 460]	449 [408; 467]	444 [427; 467]	0.23
	p-value	0.13	** < 0.001**	**0.007**	
ΔQTc in ms (N = 13)	ΔQTc_1_	0 [2; 9]	5 [3; 9]	10 [2; 17]	0.15
	ΔQTc_2_	4 [−9; 9]	7 [1; 13]	4 [−2; 13]	0.58
	ΔQTc_3_	1 [0; 9]	1 [−4; 7]	4 [−2; 9]	0.50
	ΔQTc_4_	1 [−5; 5]	2 [−1; 5]	0 [−3; 6]	0.93
	ΔQTc_last_	11 [−11; 27]	17 [7; 33]	12[−5; 26] (N = 12)	0.10
	ΔQTc_base_	12[−14; 22]	18 [6; 27]	16[3;29]	0.23
RR-interval in ms	RR_Baseline_	854 [738; 908]	869 [743; 975]	810[740; 930]	0.50
	RR_1h_	823 [745; 934]	807 [757; 978]	804 [755; 909]	0,58
	RR_2h_	781 [730; 928]	813 [719; 956]	819 [740; 916]	0.74
	RR_3h_	794 [703; 948]	805 [730; 938]	830 [753; 952]	0.20
	RR_4h_	799 [696; 950]	794 [712; 926]	827 [782; 920]	0.37
	p-value	0.58	**0.02**	0.56	

Data are expressed as median with [25th; 75th percentile]. p-value is calculated with Friedman test between and within dialysates.

*A1.50* acetic-acid dialysate with calcium concentration 1.50 mmol/l, *A1.25* acetic-acid dialysate with calcium concentration 1.25 mmol/l, *C1.50* citric-acid dialysate with calcium concentration 1.50 mmol/l, *QTc*_*Baseline*_ QTc of the first median complex (first 2 min of haemodialysis). *QTc*_*1h*_*, QTc*_*2h*_*, QTc*_*3h*_*, QTc*_*4h*_ median QTc- of respectively the first, second, third and fourth hour of haemodialysis, *ΔQTc*_*1*_*, ΔQTc*_*2*_*, ΔQTc*_*3*_*, ΔQTc*_*4*_ delta QTc of respectively between the first hour and baseline, second and first, third and second, and fourth and third hour of haemodialysis. ΔQTc_last_ = delta QTc between last complex and baseline, *ΔQTc*_*base*_ delta QTc between QTc of fourth hour and baseline.

Baseline QTc did not differ significantly between the three dialysis fluids. For C1.5, there was a significant increase of QTc between baseline and QTc_1h_, QTc_2h_, QTc_3h_ and QTc_4h_ (start–end p = 0.007; Table 1. Individually p = 0.01; p = 0.003; p = 0.006; not shown). For A1.25, there was also significant increase at QTc_1h_, QTc_2h_, QTc_3h_ and QTc_4h_ as compared to baseline (p = 0.002; p = 0.005; p = 0.009; p = 0.006 respectively; not shown). Furthermore, QTc_2h_ and QTc_3h_ significantly increased from QTc_1h_ (p = 0.02 and p = 0.04; not shown).

There were no significant differences in QTc between the various hours of HD for A1.5. For C1.5 the largest QTc change was between QTc_Baseline_ and QTc_1h_. For A1.25 the largest QTc change was between QTc_1h_ and QTc_2h_, and the second biggest QTc change was found between QTc_Baseline_ and QTc_1h_. In general, the largest rise in QTc was observed during the first hours of dialysis and was more pronounced with C1.5 and A1.25.

### RR-interval

Only A1.25 was associated with a significant decrease of RR-interval from 869[743; 975]ms at baseline till 794[712; 926]ms at fourth hour (p = 0.02), with significant differences between RR_Baseline_ and RR_1h_, RR_2h_, RR_3h_ and RR_4h_ (p < 0.05; p = 0.03; p = 0.01). There were no significant differences in ΔRR within and between the three dialysates (not shown).

### Electrolytes

Data is summarized in Table 2.

**Table 2:** Overview of electrolytes per dialysate.

		A1.5	A1.25	C1.5	p-value
iCa in mmol/l (N = 12)	Predialysis	1.14[1.07; 1.20]	1.13 [1.09; 1.21]	1.14 [1.06; 1.21]	0.91
	Post dialysis	1.23 [1.20; 1.30]	1.12 [1.08; 1.17]	1.10 [1.01; 1.13]	** < 0.001**
	ΔiCa	0.09 [0.06; 0.13]	0.0 [−0.07; 0.02]	−0.04 [−0.08; 0.03]	** < 0.001**
	ΔQTc_last_ vs. ΔiCa (r; p)^	0.13; 0.70	−0.49; 0.09	−0.46; 0.12 (N = 12)	
Magnesium in mmol/l (N = 13)	Predialysis	0.93 [0.84; 0.96]	0.90 [0.85; 1.00]	0.89; [0.77; 0.93]	**0.01**
	Post dialysis	0.79 [0.75; 0.82]	0.78 [0.75; 0.83]	0.71 [0.68; 0.75]	** < 0.001**
	ΔMg	−0.12 [−0.19; 0.05]	−0.10 [−0.22; −0.09]	−0.16 [ −0.22; −0.08]	0.47
	ΔQTc_last_ vs. ΔMg (r;p)^	0.25, 0.41	−0.47; 0.11	−**0.6; 0.03 (N = 12)**	
Bicarbonate in mmol/L (N = 13)	Predialysis	24.0 [22–25.4]	23.9 [22.4; 26.0]	23.8 [23.0; 25.0]	0.33
	Post dialysis	28.0 [21.2; 30.0]	29.0 [26.8; 30.1]	28.0 [27.0; 29.4]	0.14
	ΔBic	4.3 [3.0; 6.5]	5.0 [2.5; 6.3]	4.4 [3.0; 5.7]	0.25
	ΔQTc_last_vs. ΔBic (r;p)^	−0.37; 0.22	−0.16; 0.61	0.17; 0.58 (N = 12)	

Data is expressed as median with with [25th; 75th percentile]. p-value is calculated with Friedman test between dialysates.

*A-Ca1.50* acetic- acid dialysate with calcium concentration 1.50 mmol/l, *A-Ca1.25* acetic-acid dialysate with calcium concentration 1.25 mmol/l, *C-Ca1.50* citric-acid dialysate with calcium concentration 1.50 mmol/l, *iCa* ionized calcium, *Mg* magnesium, *Bic* bicarbonate, *ΔiCa, ΔMg, ΔBic* are calculated as post-dialysis minus predialysis values, *ΔQTc*_*last*_ delta QTc between last complex and baseline.

^r and p are measured with Spearmans’s correlation.

There was a significant difference in ΔiCa between the three dialysis fluids (p < 0.001, Table 1). It was significantly different between A1.5 (0.09[0.06; 0.13] mmol/l) and A1.25 (0.0[−0.07; 0.02] mmol/l; p = 0.002) but as well as between A1.5 and C1.5 (−0.04[−0.08; 0.03] mmol/l; p = 0.002). There was no statistical difference between A1.25 and C1.5 for ΔiCa (p = 0.15). When all values of ΔiCa and ΔQTC were put together, we found a significant inverse relation (r = -0.353, p = 0.032). This was not found within the dialysates. There was no correlation between ΔiCa and ΔQTc. There was no statistical difference between the dialysates in ΔMg and ΔBic. There was a significant inverse correlation between ΔMg and ΔQTc (r = −0.6; p = 0.03) in C1.5.

## Discussion

Our results showed that QTc increased significantly, though slightly during C1.5 and A1.25, but not during A1.5, albeit without a significant difference in QTc between the dialysis fluids. The largest changes in QTc were seen during the first hours of treatment for C1.5 and for A1.25.

In accordance with Floccari et al^4^, we suggest that the change in QTc is most likely caused by intra- and extracellular shifts of electrolytes, which are likely most pronounced at the start of dialysis because at this point in time the concentration gradient is the largest. Genovesi et al. also found a significant increase of QTc in the first hour of low dCa (1.25 mmol/l) and low potassium (2 mmol/l)^20^. Also another study with A1.25 observed a larger mean Ca loss in dialysate and ultrafiltrate at the beginning of the treatment as compared to its end^21^. An “in vivo” and “in silico” analysis was conducted by Severi et al. showing that ventricular repolarization duration is influenced by change of serum potassium and calcium depending on the dialysate concentration^22^. Other studies also showed that concentrations of Ca and K in dialysate are associated with changes in QTc^20,23^. Also in peritoneal patients, lower dCa concentrations (1.25 mmol/l) were associated with a significant increase in the QTc interval during a single dwell in contrast to a dwell with a high dCa concentration (1.75 mmol/l), although absolute changes were small^24^.

In accordance with previous studies^2^, we found a significant inverse relation between ΔiCa and ΔQTc when all values were put together. We did not find this relation within dialysates, most likely due to the small cohort of the study. Our finding that QTc remained mainly stable in A1.5 is in conformation with other studies^2^.

Based on in silico simulations, Loewe et al. suggested that heart rate variation could be calcium dependent^25^. We only found a significant decrease in RR-interval during A1.25 which could be caused by the decrease in iCa, but this was not seen with C1.5 even though postdialysis iCa was similar.

In A1.25 and C1.5, there was an increase of QTc during dialysis. This could be due to the lower Ca concentration in dialysate, as observed in a study by Severi et al.^22^. An important factor to incorporate in choosing the right dialysate concentration is whether a neutral calcium balance can be reached^1,26^. For example, calcium-profiled haemodialysis suggested by Severi et al. might have a similar QTc interval as high dCa, but with a more negative balance^17^. It is of interest to find a model where calcium can be personalized in order to maintain a neutral balance and to minimize the calcium burden and the risk for rhythm disturbances. Eventhough QT changes were observed, no clinical arrythmia was detected during the study period.

QT-intervals were corrected using Bazett’s correction method since this is the most used correction method. However. Studies have shown an over- and underestimation of QTc Bazett at RR intervals < 1000 ms and > 1000 ms respectively^27,28^. We therefore repeated the analysis after correcting the QT-intervals using Friderica’s, Fragminham’s and Hodges’ correction methods. No different significant effects were seen after using the other correction methods, i.e. only a mild prolongation in A1.25 and C1.5 (Supplementary Tables S1–3).

We did see an inverse correlation between ΔMg and ΔQTc. The difference might additionally be caused by a decline in ionized Mg, which can also be caused due to the chelating effect of citric acid. Regrettably, we did not have the opportunity to assess ionized Mg levels in our study. Because Mg was measured in the second or third dialysis with a specific dialysate, the significant difference could be caused by the C1.5 because of the chelating effect of citrate on Mg. However, the differences in predialysis Mg could also be by chance due to the small sample size and standard laboratory differences.

A limitation of our study is its small study size and the fact that patients with significantly increased QTc at baseline were excluded. Furthermore, QTc-dispersion between leads could not be calculated since precordial ECG leads were not recorded. For future research, it might be relevant to follow-up QTc in the hours after HD because it is known that Ca may rebound up to 180 min after dialysis^29^. It might also be of added value to monitor at-risk patients with implanting devices such as Reveal LINQ for detecting arrhythmias^30,31^. We did not measure plasma K values, which would have allowed to study the interaction between the effect of changes in plasma K and iCa on QTc.

Although the effects on QTc appeared in general to be relatively minor in our study population, we suggest that this study can contribute to the clinical decision to individualize the optimal dialysate for each patient, which can be based on the calcification propensity^1^, haemodynamic stability, calcium balance, the pre-existing QTc-interval or the use of QTc-prolongating medications. Extrapolating the results of our study, the effects of dCit with dCa 1.5 on the QTc-interval and calcium mass balances are comparable to those of dAcet with a 0.25 mmol/l lower dCa which is used worldwide. In conclusion, we found that the use of C1.5 or A1.25 is associated with a significantly prolongation of QTc which was however relatively limited.

## Supplementary Information

Supplementary Tables.

## Data Availability

Data cannot be shared publicly because of privacy of research participants (*i.e.*, data contain potentially identifiable patient information). Restrictions on sharing of such data are imposed by the EU General Data Protection Regulation (GDPR). Data are available for researchers who meet the criteria for access to confidential data, on reasonable request. Data can be requested via Maastricht UMC + , Dept. of Internal Medicine, Div. of Nephrology (secretariaat.nefrologie@mumc.nl) or the principal investigator, Prof. Dr. J. P. Kooman (jeroen.kooman@mumc.nl).
