# Supplementary material for: Effect of citric-acid dialysate on the QTC-interval
Source: Sci Rep. 2021 May 10;11:9909. doi: 10.1038/s41598-021-89083-w (PMC8110800; doi:10.1038/s41598-021-89083-w)
Supplement: Supplementary file 1 — Supplementary Tables. [file 41598_2021_89083_MOESM1_ESM.pdf]

EFFECT OF CITRATE-ACID DIALYSATE ON THE QTC-INTERVAL

Karlien J ter Meulen MD, Ben JM Hermans PhD, Frank M van der Sande MD PhD, Bernard Canaud MD PhD, Constantijn JAM Konings MD PhD, Jeroen P Kooman MD PhD, Tammo Delhaas MD PhD

Supplementary Tables S1-3

	**Fridericia**	A1.5	A1.25	C1.5	p-value
QTc in ms (N=13)	$QTc_{Baseline}$	423 [399; 435]	421 [390; 438]	421 [390; 433]	0.57
	QTc_{1h}	429 [403; 435]	429 [397; 445]	428 [404; 440]	0.15
	QTc_{2h}	420 [405; 436]	429 [399; 452]	424 [409; 445]	0.29
	QTc_{3h}	425 [402; 441]	428 [399; 456]	425 [413; 451]	0.30
	QTc_{4h}	431 [404; 436]	427 [405; 459]	429 [413; 457]	0.48
	p-value	0.66	**0.006**	**<0.001**	
Δ QTc in ms (N=13)	ΔQTc_{1}	2 [-1; 9]	6 [2; 8]	10 [3; 13]	**0.004**
	ΔQTc_{2}	4 [-7; 8]	4 [0; 10]	4 [-3; 10]	0.79
	ΔQTc_{3}	1 [-1; 7]	0 [-3; 4]	4 [0; 8]	0.29
	ΔQTc_{4}	0 [-4; 5]	0 [-2; 4]	0 [-2; 5]	0.87
	ΔQTc_{last}	6 [-9; 19]	9 [1; 26]	15 [0; 25] (N=12)	0.34
	ΔQTc_{base}	8 [-5; 19]	10 [2; 24]	21 [2; 28]	0.20

Table S1. **Overview of corrected QT-interval per dialysate, calculating by method of Fridericia.** Data are expressed as median with [25th; 75th percentile]. p-value is calculated with Friedmand test between and within dialysates. A1.50 = acetic- acid dialysate with calcium concentration 1.50mmol/l. 1.25= acetic-acid dialysate with calcium concentration 1.25mmol/l. C1.50 = citric-acid dialysate with calcium concentration 1.50mmol/l. $QTc_{Baseline}$ = QTc of the first median complex (first 2 minutes of haemodialysis). QTc_{1h}, QTc_{2h}, QTc_{3h}, QTc_{4h} = median QTc- of respectively the first, second, third and fourth hour of haemodialysis. ΔQTc_{1}, ΔQTc_{2}, ΔQTc_{3}, ΔQTc_{4} = delta QTc of respectively between the first hour and baseline, second and first, third and second, and fourth and third hour of haemodialysis.

	Framingham	A1.5	A1.25	C1.5	p-value
QTc in ms (N=13)	$QTc_{Baseline}$	423 [401; 436]	422 [391; 437]	417 [393; 431]	0.46
	QTc_{1h}	428 [404; 435]	427 [394; 444]	422 [405; 440]	0.20
	QTc_{2h}	420 [407; 436]	426 [397; 452]	420 [410; 444]	0.29
	QTc_{3h}	425 [403; 439]	424 [397; 455]	426 [410; 449]	0.23
	QTc_{4h}	429 [405; 435]	422 [403; 457]	428 [409; 455]	0.48
	p-value	0.74	**0.018**	**0.001**	
Δ QTc in ms (N=13)	ΔQTc_1	3 [-1; 8]	5 [1; 8]	9 [2; 13]	**0.002**
	ΔQTc_2	4 [-7; 8]	4 [0; 9]	4 [-4; 9]	0.58
	ΔQTc_3	1 [-2; 4]	0 [-4; 3]	4 [0; 6]	0.29
	ΔQTc_4	0 [-4; 4]	1 [-2; 3]	0 [-2; 5]	0.84
	ΔQTc_{last}	4 [-10; 16]	9 [0; 25]	15 [0; 25] (N=12)	0.34
	ΔQTc_{base}	6 [-7; 17]	10 [-1; 23]	19 [2; 28]	0.29

Table S2. Overview of corrected QT-interval per dialysate, calculating by method of Framingham. Data are expressed as median with [25th; 75th percentile]. p-value is calculated with Friedmand test between and within dialysates. A1.50 = acetic- acid dialysate with calcium concentration 1.50mmol/l. 1.25= acetic-acid dialysate with calcium concentration 1.25mmol/l. C1.50 = citric-acid dialysate with calcium concentration 1.50mmol/l. $QTc_{Baseline}$ = QTc of the first median complex (first 2 minutes of haemodialysis). QTc_{1h}, QTc_{2h}, QTc_{3h}, QTc_{4h} = median QTc- of respectively the first, second, third and fourth hour of haemodialysis. ΔQTc_1, ΔQTc_2, ΔQTc_3, ΔQTc_4 = delta QTc of respectively between the first hour and baseline, second and first, third and second, and fourth and third hour of haemodialysis.

	**Hodges**	A1.5	A1.25	C1.5	p-value
QTc in ms (N=13)	$QTc_{Baseline}$	421 [401; 433]	418 [389; 438]	419 [390; 432]	0.78
	QTc_{1h}	426 [403; 431]	427 [398; 443]	431 [402; 440]	0.37
	QTc_{2h}	421 [404; 431]	427 [405; 449]	435 [408; 446]	0.5
	QTc_{3h}	425 [403; 439]	429 [403; 452]	430 [411; 454]	0.06
	QTc_{4h}	429 [404; 436]	425 [405; 455]	426 [412; 453]	0.32
	p-value	0.45	**0.002**	**<0.001**	
Δ QTc in ms (N=13)	ΔQTc_1	3 [-1; 10]	5 [3; 9]	7 [2; 13]	**0.01**
	ΔQTc_2	3 [-6; 8]	4 [1; 9]	5 [0; 11]	0.43
	ΔQTc_3	1 [-1; 7]	0 [-3; 4]	4 [3; 7]	0.37
	ΔQTc_4	0 [-5; 6]	1 [-1; 3]	0 [-2; 4]	0.74
	ΔQTc_{last}	9 [-9; 23]	8 [3; 25]	15 [2; 25] (N=12)	0.34
	ΔQTc_{base}	9 [-5; 24]	8 [4; 24]	17 [5; 31]	0.09

Table S3. Overview of corrected QT-interval per dialysate, calculating by method of Friderica. Data are expressed as median with [25th; 75th percentile]. p-value is calculated with Friedmand test between and within dialysates. A1.50 = acetic- acid dialysate with calcium concentration 1.50mmol/l. 1.25= acetic-acid dialysate with calcium concentration 1.25mmol/l. C1.50 = citric-acid dialysate with calcium concentration 1.50mmol/l. $QTc_{Baseline}$ = QTc of the first median complex (first 2 minutes of haemodialysis). QTc_{1h}, QTc_{2h}, QTc_{3h}, QTc_{4h} = median QTc- of respectively the first, second, third and fourth hour of haemodialysis. ΔQTc_1, ΔQTc_2, ΔQTc_3, ΔQTc_4 = delta QTc of respectively between the first hour and baseline, second and first, third and second, and fourth and third hour of haemodialysis.
